# Constructing small genome graphs via string compression

**DOI:** 10.1093/bioinformatics/btab281

**Published:** 2021-07-12

**Authors:** Yutong Qiu, Carl Kingsford

**Affiliations:** Computational Biology Department, School of Computer Science, Carnegie Mellon University, Pittsburgh, PA 15213, USA; Computational Biology Department, School of Computer Science, Carnegie Mellon University, Pittsburgh, PA 15213, USA

## Abstract

**Motivation:**

The size of a genome graph—the space required to store the nodes, node labels and edges—affects the efficiency of operations performed on it. For example, the time complexity to align a sequence to a graph without a graph index depends on the total number of characters in the node labels and the number of edges in the graph. This raises the need for approaches to construct space-efficient genome graphs.

**Results:**

We point out similarities in the string encoding mechanisms of genome graphs and the external pointer macro (EPM) compression model. We present a pair of linear-time algorithms that transform between genome graphs and EPM-compressed forms. The algorithms result in an upper bound on the size of the genome graph constructed in terms of an optimal EPM compression. To further reduce the size of the genome graph, we propose the source assignment problem that optimizes over the equivalent choices during compression and introduce an ILP formulation that solves that problem optimally. As a proof-of-concept, we introduce RLZ-Graph, a genome graph constructed based on the relative Lempel–Ziv algorithm. Using RLZ-Graph, across all human chromosomes, we are able to reduce the disk space to store a genome graph on average by 40.7% compared to colored compacted de Bruijn graphs constructed by Bifrost under the default settings. The RLZ-Graph scales well in terms of running time and graph sizes with an increasing number of human genome sequences compared to Bifrost and variation graphs produced by VGtoolkit.

**Availability:**

The RLZ-Graph software is available at: https://github.com/Kingsford-Group/rlzgraph.

**Supplementary information:**

Supplementary data are available at *Bioinformatics* online.

## 1 Introduction

The linear reference genome suffers from reference bias that results in discarding informative reads sequenced from nonreference alleles during alignment (Ballouz *et al.*, 2019). To reduce the reference bias, alternative read alignment approaches that use a set of genomes as the reference have been recently introduced (Chen *et al.*, 2020; Novak *et al.*, 2017). Genome graphs, due to their compact structure to store the shared regions of highly similar strings, are widely used to represent and analyze a collection of genomes compactly (Computational Pan-Genomics Consortium, 2018; Paten *et al.*, 2017; Sherman and Salzberg, 2020).

A genome graph of a collection of sequences is a labeled directed multigraph such that each sequence is equal to the concatenation of node labels on a path. We call such a path a reconstruction path. The size of a genome graph is the space to store the graph structure, which is the set of nodes, edges and node labels.

The size of a genome graph is crucial to the efficiency of operations such as mapping sequencing reads. As shown in Jain *et al.* (2019), the time complexity of mapping a string to a genome graph is directly related to the total number of characters in node labels and the number of edges. The speed of sequence-to-graph mapping can be further improved by a graph index, the size of which is also dependent on the size of the genome graphs (Paten *et al.*, 2017; Sirén *et al.*, 2014, 2020).

Most of the existing genome graph construction algorithms do not directly optimize the size of the genome graph. Some of these algorithms design graph structures to adapt to specific types of input data, such as read alignment (Garrison *et al.*, 2018; Li *et al.*, 2020; Paten *et al.*, 2011; Mäkinen *et al.*, 2020), variant calls (Garrison *et al.*, 2018; Rakocevic *et al.*, 2019; Dilthey *et al.*, 2015) or raw sequencing reads (Iqbal *et al.*, 2012; Li *et al.*, 2020), which are not necessarily optimized. Others only optimize the graph index that stores reconstruction paths based on assumed types of genome graphs, for example, the variation graphs (Sirén, 2017; Sirén *et al.*, 2020) or the colored compacted de Bruijn graphs (Almodaresi *et al.*, 2017, 2020; Holley and Melsted, 2020; Minkin *et al.*, 2017; Muggli *et al.*, 2019). As a result, the graphs constructed can be large in terms of both the space taken by the graph structure or the lengths of the reconstruction paths.

While a small genome graph is desirable, the smallest genome graph may be useless if each edge is allowed to be traversed multiple times. The smallest genome graph is a multigraph with four nodes, or *G*_4_, whose labels are *A*, *T*, *C* and *G*, respectively. The edges in *G*_4_ are two edges directing to and from each pair of nodes and a self-loop on each node. *G*_4_ contains the reconstruction path for any sequence over alphabet {A,T,C,G}. However, the length of the reconstruction paths of each string would have lengths equal to the lengths of the string, which undermines the goal of a genome graph to compactly store similar strings.

In order to construct a small genome graph that balances the size of the graph and the lengths of the reconstruction paths, we introduce the definition of a restricted genome graph and formalize the restricted genome graph optimization problem, which seeks to build the smallest restricted genome graph given a collection of strings.

We present a genome graph construction algorithm that directly addresses the restricted genome graph size optimization problem. Optimizing the size of a restricted genome graph is similar to optimizing the space taken by a set of strings, which echoes the external pointer macro (EPM) compression scheme (Storer, 1977). We introduce a pair of algorithms that transform between the EPM-compressed form and the restricted genome graphs and prove an upper bound on the size of the restricted genome graph constructed given an optimized EPM-compressed form from a set of input sequences. We further reduce the number of nodes and edges by introducing and solving the source assignment problem via integer linear programming (ILP).

As a proof-of-concept that compression-based genome graph construction algorithms produce small genome graphs efficiently, we build the RLZ-Graph, which is based on an EPM compression heuristic known as the relative Lempel–Ziv (RLZ) algorithm. The EPM compression problem is NP-complete (Storer and Szymanski, 1982). Among the approximation heuristics to solve the EPM compression problem, the RLZ algorithm (Kuruppu *et al.*, 2010) runs in linear time and achieves good compression ratios on human genomic sequences (Deorowicz *et al.*, 2015; Deorowicz and Grabowski, 2011; Ferrada *et al.*, 2014). The RLZ algorithm is based on the Lempel–Ziv (LZ) algorithm (Ziv and Lempel, 1977). Although the LZ algorithm achieves a better compression ratio than RLZ, it does not follow the EPM scheme and thus is not applicable in EPM-based genome graph construction.

We evaluate the performance of the RLZ-Graph by comparing to the colored compacted de Bruijn graphs (ccdBG) (Iqbal *et al.*, 2012). CcdBG construction methods, similar to the compression-based genome graph construction algorithms, process the input sequences directly without intermediate steps such as alignment or variant calling. In ccdBG, the input sequences are fragmented into preliminary nodes that represent unique strings of length *k*, or *k*-mers. Each edge represents the adjacency between two *k*-mers in the sequences stored. The preliminary nodes with in- and out-degrees equal to 1 on a path are further merged into supernodes. Still, the number of nodes and edges, as well as the number of characters in node labels, in a ccdBG can increase significantly as the number of sequences stored increases. The size of the graph also depends heavily on the parameter *k*. These factors may offset the effort to efficiently encode the reconstruction path information in the graph indices (Almodaresi *et al.*, 2020, 2017; Holley and Melsted, 2020; Minkin *et al.*, 2017; Muggli *et al.*, 2019). Despite the different approaches to build the ccdBG indices, ccdBG construction methods result in the same underlying de Bruijn graph structure. When we compare our algorithm with ccdBG construction algorithms, we only compare the graph structure, which includes nodes, edges and sequences stored in each node.

The performance of RLZ-Graph is examined in multiple aspects. We show that the RLZ-Graph scales well in terms of running time and graph sizes with a large number of genomic sequences by comparing sizes of the RLZ-Graph with the ccdBG constructed by Bifrost (Holley and Melsted, 2020) from 100 individuals on all chromosomes from the 1000 Genomes Project (1000 Genomes Project Consortium, 2015) (Section 8.1). Across all chromosomes, the disk space taken to store the graph representation of 100 sequences is on average reduced by 40.7% compared with the ccdBG built under the default settings. Additionally, we examine the performance of RLZ-Graph, Bifrost and VGtoolkit (Garrison *et al.*, 2018) on 32 individuals from the HGSVC dataset (Ebert *et al.*, 2021) that contains more complex structural variants than the 1000 Genomes Project samples (Section 8.2). On this dataset, the advantage of RLZ-Graph over ccdBGs persists, and RLZ-Graph constructs genome graphs of similar sizes as variation graphs constructed by VGtoolkit.

Additionally, we evaluate the performance of the ILP solution to the source assignment problem on RLZ-Graphs constructed from *E. coli* genome sequence. We show that the solutions to the source assignment problem reduce the number of nodes by around 8% on 300 *E. coli* genomes (Clark *et al.*, 2016) (Supplementary Section S3).

## 2 Definitions

### 2.1 Strings

Let *s* be a string. s[b:e] denotes a substring starting from position *b* (inclusively) of *s* up to position *e* (inclusively). We assume 0-indexing throughout this article. The length of *s* is denoted by |s|. Concatenation of strings $\left\{s_{1},\ldots ,s_{n}\right\}$ is denoted by $s\prime =s_{1}\cdot s_{2}\cdot \ldots \cdot s_{n}$.

### 2.2 Genome graphs

Definition 1 (Genome graph). *A genome graph* G=(V,E,ℓ)  *of a collection of strings* $S=\{s_{1},s_{2},\ldots ,s_{n}\}$  *is a directed multigraph with node set V, edge multiset E, and node labels* ℓ(u)  *for each node u. A genome graph of* S  *contains a collection of paths* $P=\{P_{1},P_{2},\ldots ,P_{n}\}$*, where* $P_{i}=v_{1},v_{2},\ldots ,v_{\left|P_{i}\right|}$*, such that* $s_{i}=\ell (v_{1})\cdot \ell (v_{2})\cdot \ldots \cdot \ell (v_{\left|P_{i}\right|})$  *for all* $s_{i}\in S$*. Such paths are called reconstruction paths.*

The size of a genome graph G=(V,E,ℓ) is denoted by *size*(*G*), which is the space to store the set of nodes, edges and node labels (Section 3.1). The number of nodes in the node set *V* and the number of edges in edge multiset *E* are denoted as |V| and |E|, respectively. A genome graph may contain parallel edges, where two or more edges are incident to the same pair of nodes, and each edge may be traversed multiple times. We introduce the notion of a restricted genome graph, which limits the number of traversals through each edge.

Definition 2 (Restricted genome graph). *A restricted genome graph is a genome graph with a source and sink node and the restriction that each edge is allowed to be traversed at most once in all reconstruction paths. A source is a node with no incoming edges, which represents the start of all stored sequences. A sink is a node with no outgoing edges, which represents the end of all stored sequences.*

**Fig. 1:**
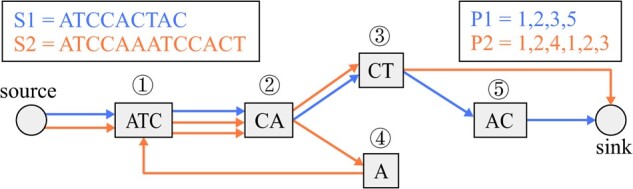
An example of a restricted genome graph. The graph stores two strings, S1 and S2. The color of the edges denotes the origin of node adjacencies. The reconstruction paths for S1 and S2 are shown in the upper right box.

An example of a restricted genome graph is shown in Figure 1. Each edge is traversed only once in all reconstruction paths, and parallel edges are present. In a restricted genome graph, if we add edges directing from sink to source, then the concatenation of all reconstruction paths forms an Eulerian tour. For a restricted genome graph G=(V,E,ℓ) and a collection of all reconstruction paths $P=\{P_{1},P_{2},..,P_{n}\}$, we have $|E|=\sum_{P_{i}\in P}\left(|P_{i}|-1\right)+2n$, where 2*n* edges are the edges directing from the source and edges directing to the sink.

### 2.3 EPM compression scheme

We review the definition of the EPM scheme for data compression (Storer and Szymanski, 1982).

Definition 3 (Pointers in EPM). *Given a reference string R, a pointer* $p_{i}=(pos_{i},len_{i})$  *represents the substring* $R[pos_{i}:pos_{i}+len_{i}-1]$.

We say that two pointers, $p_{i}=(pos_{i},len_{i})$ and $p_{j}=(pos_{j},len_{j})$ are equivalent to each other if $R[pos_{i}:pos_{i}+len_{i}-1]=R[pos_{j}:pos_{j}+len_{j}-1]$. We refer to the length of a pointer $p_{i}=(pos_{i},len_{i})$ as $p_{i}.\mathrm{len}$ and the position of a pointer as $p_{i}.\mathrm{pos}$.

Definition 4 (External pointer macro (EPM) model (Storer and Szymanski, 1982)). *Given an alphabet Σ and a string T, a compressed form of string T adopts the EPM model if the compressed data follows the form* C=R#t*, where R is a string over Σ,* $t=p_{1}p_{2}\ldots$  *is a sequence of pointers that represent substrings in R,* #  *is a separator symbol that is not in Σ, and T is equal to the string produced by substituting pointers in t by their corresponding substrings.*

The string *T* may represent a set of strings $S=\{s_{1},s_{2},\ldots ,s_{n}\}$ by concatenation, i.e., *T* = *s*_1_$$s_{2}\ldots$$*s_n_*, where $≠# and $∉Σ, where Σ is the alphabet for *S*. In order to define the end of each string and forbid pointers to cross the boundaries between strings, the alphabet for *T* is be constructed as $\Sigma `=\Sigma \cup \left\{\$\right\}$. Additionally, a $ character is appended to the end of the reference string.

It is natural to consider optimizing the size of the compressed string *C*, *size*(*C*) (Section 3.2), which leads to:

Problem 1 (EPM decision problem (Storer and Szymanski, 1982)). *Given a string T and an integer m, determine if there is a compressed form C of T that follows EPM such that* size(C)≤m.

In Storer and Szymanski (1982), Problem 1 is shown to be NP-complete.

In EPM, the substring represented by a pointer may occur several times in the reference string. We define such occurrences as sources of a pointer:

Definition 5 (Source). *A source,* $\left(pos_{1},\mathrm{len}\right)$*, of a pointer* $p=(pos_{2},\mathrm{len})$  *is an occurrence of* $R[pos_{2}:pos_{2}+\mathrm{len}-1]$  *in R. In other words,* $R[pos_{1}:pos_{1}+\mathrm{len}-1]=R[pos_{2}:pos_{2}+\mathrm{len}-1]$*. Each pointer p is associated with a source set* $S_{p}=\{ss_{1},ss_{2},\ldots \}$*, where* $R[ss_{i}.\mathrm{pos}:ss_{i}.\mathrm{pos}+ss_{i}.\mathrm{len}-1]=R[p.\mathrm{pos}:p.\mathrm{pos}+p.\mathrm{len}-1]$  *for all* $ss_{i}\in S_{p}$.

Sources are used to refer to the occurrences of a substring on the reference string *R*, and pointers are used to refer to the pair of integers eventually stored in the compressed string *t*.

Definition 6 (Boundaries of sources and pointers). *The boundaries of a source* s=(pos,len)  *are defined as (b, e), where and* e=pos+len*. b is the left boundary and e is the right boundary. The similar definition of boundaries applies to pointers.*

Two boundaries, (*b*_1_, *e*_1_) and (*b*_2_, *e*_2_), intersect if and only if *b*_1_ = *b*_2_ or *b*_1_ = *e*_2_ or *e*_1_ = *b*_2_ or *e*_1_ = *e*_2_.

### 2.4 RLZ algorithm

Definition 7 (Phrase). *Given a reference string R and a string T, let pointer* p=(pos,len)  *represent the substring* R[pos:pos+len−1]  *which equals* T[pos′:pos′+len−1]  *for some position* pos′*. Then p is a phrase if p is right-maximal, i.e. if* R[pos:pos+len]≠T[pos′:pos′+len].

The RLZ algorithm, proposed by Kuruppu *et al.* (2010), runs in linear time and achieves good compression ratios with genomic sequences. The RLZ algorithm takes a reference string *R* as input and parses the input string *T* greedily from left to right. At position *i* in *T*, the RLZ algorithm substitutes the longest prefix of T[i:|T|−1] that matches a substring in *R* with a phrase. Let the length of the phrase be *len*. After substitution, the RLZ algorithm skips to position i+len in *T* and repeats the substitution process until *T* is exhausted. The process of phrase production is called RLZ factorization. In some analyses of the RLZ algorithm, the reference string is generated from the set of input strings (Gagie *et al.*, 2016). Nevertheless, the RLZ algorithm given a reference string remains the same.

**Fig. 2:**
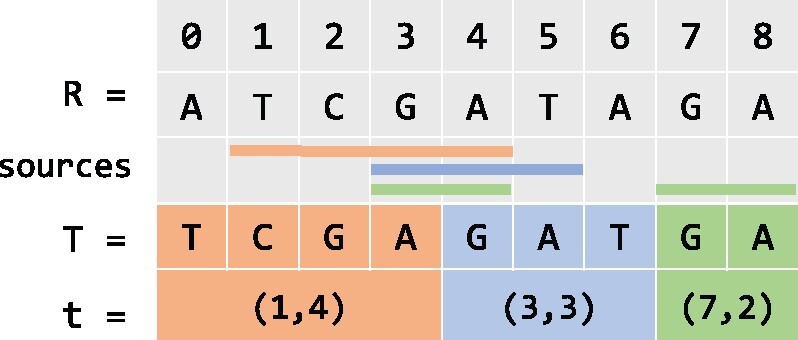
An example of RLZ factorization. The top row is the indices of characters in the strings. *R* is the reference string, *T* is the input string and *t* is a sequence of phrases resulted from RLZ factorization. Colored line segments on the third row represent the sources associated with phrases with the same color

The definitions introduced above are demonstrated in Figure 2, where *R* is the reference string and *T* is the input string to the RLZ algorithm. RLZ factors *T* into a sequence of three phrases, shown as *t*. The compressed form of the input string *T* is C=R#t. Each phrase is associated with some sources that are represented as line segments in the figure. For example, the last phrase, (7, 2), replaces the substring T[7:8]. It also corresponds to two sources in *R*: (3, 2) and (7, 2), which are represented by the green line segments in *R*. The left and right boundaries of phrase (7, 2) are (b=7,e=8) in *T*. Source (3, 2) intersects with sources (1, 4) and (3, 3). However, sources (1, 4) and (3, 3) do not intersect with each other.

## 3 Size formulation of restricted genome graphs and EPM-compressed forms

### 3.1 Size of a genome graph

We adopt a natural formulation of the size of a labeled graph, which describes the space to store nodes, edges and node labels. Given a restricted genome graph G=(V,E,ℓ) over alphabet Σ, let *L* be a string that contains every node label as a substring. Each node can be represented as a pointer to *L*, i.e., v=(pos,len), such that ℓ(v)=L[pos:pos+len−1]. Each node takes 2 log |L| bits to be stored. The edges are stored as pairs of adjacent nodes. Each edge takes space 2 log |V| bits. Therefore, the total space taken by a restricted genome graph, denoted by *size*(*G*), under this model is:

(1) size(G)=|L|· log |Σ|+|V|·2 log |L|+|E|·2 log |V|.

We introduce the restricted genome graph optimization problem:

Problem 2 (Restricted genome graph optimization problem). *Given a set of sequences, build a restricted genome graph G such that size(G) is minimized.*

In the above formulation, note that |E| refers to the number of edges including the parallel edges. Solutions to Problem 2 avoid a trivial genome graph solution, that is a multigraph, *G*_4_, with four nodes whose labels are *A*, *T*, *C* and *G*, respectively, and edges such that there are at least two edges with different directions between each pair of nodes. Any sequence over the alphabet Σ={A,T,C,G} can be reconstructed in *G*_4_ under the definition of a genome graph (Definition 1) since each edge may be traversed more than once. On the other hand, in a restricted genome graph, the number of edges grows as the lengths of reconstruction paths increase. Therefore, minimizing the size of the restricted genome graph achieves a combined objective of a small genome graph and short parsing of the input sequences.

### 3.2 Size of an EPM-compressed form

Next, we quantify the space taken by an EPM-compressed form C=R#t. The space taken by *C*, *size*(*C*), is the space to store the total number of unique pointers in *t*, the sequence *t* and the reference string *R*. We first encode each unique pointer with a pair of integers, (*pos*, *len*), which takes space 2 log |R| bits. If there are *n* unique pointers, *t* can be stored as a sequence of identifiers of the unique pointers using |t| log n bits. Therefore, the total space taken by an EPM-compressed form is

(2) size(C)=|R|· log |Σ|+|t|· log n+n·2 log |R|.

From equations (1) and (2), both the restricted genome graph and the EPM-compressed form have a size formulation that has three terms, which are the space taken by a reference string, the space taken by the unique pointers and the space to store the adjacencies between pointers.

In order to reduce the size of the restricted genome graphs (Definition 2), it is natural to borrow ideas from the field of string compression. We introduce two algorithms that transform between genome graphs and compressed strings produced by EPM compression scheme (Storer and Szymanski, 1982).

## 4 Transformation between EPM-compressed forms and genome graphs

In the following sections, we assume that the input string, *T*, is a concatenation of a set of sequences and the sequences are separated by the special symbol $.

### 4.1 EPM-compressed string to genome graph

Given an EPM-compressed form C=R#t of the original string *T*, and an alphabet Σ, the genome graph construction algorithm produces a restricted genome graph that stores both *R* and *T*.

**Fig. 3:**
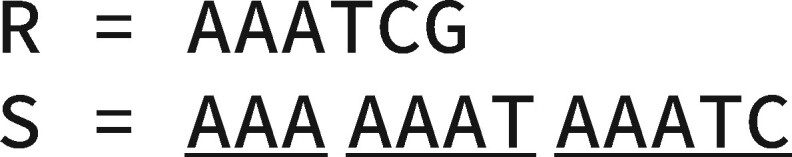
String *S* is factored into three pointers given the reference string *R*. Each underlined substring is represented by a different pointer. According to the naïve algorithm to construct the genome graph, three nodes are created from three pointers

A naïve algorithm to construct a genome graph is to create a node for each unique pointer in *t* and add an edge between nodes that represent each pair of pointers t[i] and t[i+1]. However, in repetitive sequences such as the human genome, a substring may occur in several pointers and thus may be stored several times redundantly. As shown in Figure 3, the substring AAA would be stored three times according to the naïve algorithm, which results in excess space spent on storing repetitive content.

Our construction algorithm, introduced below as two-pass CtoG, merges the repetitive substrings shared by multiple pointers by grouping pointers by their positions on the reference. Two-pass CtoG constructs the genome graph in two passes through *t*. In the first pass, the algorithm creates nodes by cutting the reference string according to the boundaries of each pointer. In the second pass, the algorithm connects the nodes according to the adjacencies between pointers in the compressed string *t*.

**First pass.**

Create a bit vector, B. A bit set at B[i] indicates that a pointer boundary falls at position *i* on *R*. Process *t* from left to right. For each pointer p=(pos,len), mark its boundaries in B: B[pos]=1 and B[pos+len]=1. After *t* is exhausted, transform B into a succinct bitvector that supports rank operations in constant time (e.g. Kärkkäinen *et al.*, 2014; Raman *et al.*, 2002), where rank${}_{B}(i)$ returns the number of set bits at or before position *i* in B. We then cut the reference string at positions where a bit is set in B. If B[i] and B[j] are the only set bits in the interval [i:j], we create a node v=(pos,len)=(i,j−i) with ℓ(v)=R[i:j−1]. Each node can be treated as a pointer whose left and right boundaries are *i* and *j*, respectively. Each node is identified using its left boundary, i.e., rank${}_{B}(i)$.

We define the ordering of nodes: $v_{i}=(pos_{i},len_{i})≺v_{j}=(pos_{j},len_{j})$ and *i *<* j* iff *pos_i_* < *pos_j_*, where *i* and *j* are the identifiers of *v_i_* and *v_j_*. Since nodes are created by cutting the reference, different nodes will always have different starting positions. Add an edge between each *v_i_* and $v_{i+1}$ for all i<|V|−1. The path $v_{1},v_{2},\ldots ,v_{\left|V\right|}$ represents the reference string *R*.

**Second pass.**

We process *t* from left to right again in the second pass.

Create a source and a sink node that represent the start and end of each sequence represented in *T*. Add an edge to the node whose position corresponds to t[0].

For each pointer t[i], add reference edges between nodes that fall between the range of *pos_i_* and $pos_{i}+len_{i}$, i.e., nodes in $\left\{v_{j}|pos_{i}\leq v_{j}.\mathrm{pos}<pos_{i}+len_{i}\right\}$. The identifiers of such nodes are consecutive because the nodes are sorted by their starting positions. The range of such node identifiers is between rank${}_{B}(pos_{i})$ and rank${}_{B}(pos_{i}+len_{i}-1)$. If a pointer $t[i]=(pos_{i},len_{i})$ represents the suffix of a sequence, then $R[pos_{i}+len_{i}-1]=$ $. When such pointer is encountered, add an edge from node $v_{\mathrm{ran}k_{B}(pos_{i}+len_{i}-1)}$ to sink. Add an edge from the source to node $v_{\mathrm{ran}k_{B}(t[i+1].\mathrm{pos})}$.

For each pair of pointers t[i] and t[i+1], we need to connect the nodes that mark the right and left boundaries of t[i] and t[i+1], respectively. Let $t[i]=(pos_{i},len_{i})$ and $t[i+1]=(pos_{i+1},len_{i+1})$. We need to find two nodes, $v_{m}=(pos_{m},len_{m})$ and $v_{n}=(pos_{n},len_{n})$, such that $pos_{m}+len_{m}=pos_{i}+len_{i}$ and $pos_{n}=pos_{i+1}$. Since each node is identified by their left boundary, two nodes can be identified by $m=\mathrm{ran}k_{B}(pos_{i}+len_{i}-1)$ and $n=\mathrm{ran}k_{B}(pos_{i+1})$. Edge (*v_m_*, *v_n_*) then represents the adjacency between t[i] and t[i+1] in *t*.

**Fig. 4:**
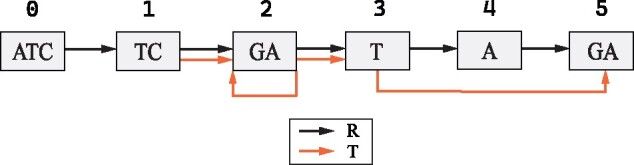
The RLZ-Graph of reference *R* = *ATCGATAGA* and input string *T* = *TCGAGATGA* (Fig. 2). The black path 0,1,2,3,4,5 encodes *R*, the orange path 1,2,2,3,5 encodes *T*. The parallel edges are shown for the purpose of illustration and are merged in the final graph

Repeat the process until *t* is exhausted. An example output of the algorithm is shown in Figure 4, where the compressed string is produced by the RLZ algorithm (Kuruppu *et al.*, 2010).

The running time of the construction algorithm is O(|T|+|R|). In the first pass, the algorithm passes through each pointer once, and the number of pointers, |t|≤|T|. The number of created nodes is at most |R|. In the second pass, the algorithm adds at most |T| reference edges and |t| edges that represent adjacency between pointers. In the actual implementation, the parallel edges are merged. Therefore, during the second pass, we do not need to add reference edges for each pointer. Hence, the running time in the actual implementation is O(|t|+|R|).

The constructed restricted genome graph stores the set of nodes, edges and node labels. While storing the reconstruction paths is also important, it is a separate challenge from optimizing the graph structure. There has been a line of work that constructs small graph indices to store the reconstruction paths efficiently given any graph structure (Sirén *et al.*, 2014, 2020; Sirén, 2017). These indices can also be applied to our genome graph.

There are three types of edges in the produced restricted genome graph: the backward edges, the forward edges and the reference edges. We define the backward edges as edges that direct from *v_j_* to *v_i_*, where j≥i, which include self-loops. We define the forward edges as edges that direct from *v_i_* to *v_j_*, where i<j−1. We define the reference edges as the edges that direct from *v_i_* to $v_{i+1}$. In other words, reference edges $\left(v_{i}=(pos_{i},len_{i}),v_{j}=(pos_{j},len_{j})\right)$ connect nodes where the first node’s right boundary intersects with the second node’s left boundary, i.e. $pos_{i}+len_{i}=pos_{j}$.

We show that the constructed graph is a restricted genome graph that contains reconstruction paths for *R* and *T* as in Theorem 1, for which the detailed proof can be found in the Supplementary material.

Theorem 1. *Given an EPM-compressed form of string T,* C=R#t*, the algorithm described above creates a genome graph* G=(V,E,ℓ)  *that contains reconstruction paths for R and T.*

### 4.2 Genome graph to EPM-compressed form

Given a restricted genome graph G=(V,E,ℓ) and a set of reconstruction paths P that represent strings in S, we present an algorithm, GtoC, that produces an EPM-compressed form C=R#t whose decompression equals string *T*, which is a concatenation of strings in S.

Produce the reference string *R* by concatenating the node labels in an arbitrary order. Each node can then be represented as a pointer to *R* and be denoted as $v_{i}=(pos_{i},len_{i})$, where $\ell (v_{i})=R[pos_{i}:pos_{i}+len_{i}-1]$. Assign an identifier to each node such that for *v_i_* and *v_j_*, *i *<* j* if *pos_i_* < *pos_j_*.

Process all P∈P by substituting nodes with their pointer representations. If two or more adjacent nodes $v_{i},v_{i+1},\ldots ,v_{j}$ in *P* are connected by a reference edge, merge the two nodes into one pointer $p=(pos_{i},pos_{j}+len_{j}-pos_{i})$. Concatenate all processed *P*, which results in *t*. The converted sequence of pointers *t* is then $p_{1},p_{2},\ldots ,p_{\left|t\right|}$, where $\left|t|\leq \sum_{P\in P}|P\right|$.

The converted *C* satisfies the EPM definition where *R* is a string over Σ, and *t* is a sequence of pointers to substrings in *R*. Since the concatenation of paths in P spells out *T* by concatenating all the labels of nodes on the path, substituting the pointers in *t* with corresponding substrings reconstructs *T*.

The running time of the GtoC construction algorithm is $O(|V|+\sum_{P\in P}|P|)=O(|V|+|E|)$.

The size of the produced EPM-compressed form can be further reduced if the reference string *R* is equal to the shortest superstring that contains all the node labels. While finding the shortest superstring problem is NP-hard (Räihä and Ukkonen, 1981) when the number of nodes is greater than 2, it may be approached by using approximation algorithms (Alanko and Norri, 2017; Blum *et al.*, 1994; Turner, 1989).

## 5 Upper-bound on the size of the restricted genome graph and the EPM-compressed form

We show that the size of a restricted genome graph *G* produced using the two-pass CtoG algorithm is bounded by the terms of the input EPM-compressed form *C* (Lemma 1). In the following lemmas, we assume that the input string *T* represents a concatenated set of *m* sequences. The details of the proofs are in the Supplementary material.

Lemma 1. *Given an optimally compressed EPM form* C=R#t  *of text T, the size of the transformed restricted genome graph* G=(V,E,ℓ)*, size(G), according to two-pass CtoG in Section 4.1 has an upper bound:*
 

(3) $$\begin{array}{c} s\mathrm{ize}(G)\leq |R|\cdot \;\text{log}\;|\Sigma |+\min(2n,|R|)\cdot 2\;\text{log}\;|R|\\ +(\min(2n,|R|)\cdot |t|-1+2m)\cdot 2\;\text{log}(\min(2n,|R|)) \end{array}$$,

*where n is the number of unique pointers in t.*

In practice, the graphs are stored such that the parallel edges are merged. We show that the size of the genome graph G′ produced by merging the parallel edges in *G* can also be bounded by the terms of the EPM-compressed form *C* (Lemma 2).

Lemma 2. *Given a restricted genome graph,* G=(V,E,ℓ)*, constructed from an optimally compressed EPM form* C=R#t*, the size of the genome graph,* G′=(V,E′,ℓ)*, produced by merging parallel edges in G has an upper bound:*
 

(4) $$\begin{array}{c} s\mathrm{ize}(G\prime )\leq |R|\cdot \;\text{log}\;|\Sigma |+\min(2n,|R|)\cdot 2\;\text{log}\;|R|\\ +(\min(2n,|R|)+|t|-1+2m)\cdot 2\;\text{log}(\min(2n,|R|)), \end{array}$$

*where n is the number of unique pointers in t.*

We show in Lemma 3 that the size of the EPM-compressed form produced by GtoC algorithm (Section 4.2) is upper-bounded by the terms of the size of a restricted genome graph.

Lemma 3. *Given a restricted genome graph* G=(V,E,ℓ)  *of a collection of strings* S*, the size of the transformed EPM-compressed form of the concatenated strings in* S, C=R#t  *according to GtoC described in Section 4.2 has an upper bound:*
 

(5) $$\begin{array}{c} s\mathrm{ize}(C)\leq |R|\cdot \;\text{log}\;|\Sigma |+|E|\cdot \;\text{log}\;\left(\begin{array}{c} |V|+1\\ 2 \end{array}\right)\\ +2\left(\begin{array}{c} |V|+1\\ 2 \end{array}\right)\;\text{log}\;|R|, \end{array}$$

*where R is a string formed by concatenating all node labels.*

The pair of algorithms do not produce an optimal genome graph or optimal EPM-compressed form. Still, given an optimal input, the algorithms achieve results that are bounded by the original terms in the input. We further improve the transformation from EPM-compressed form to genome graph by addressing the source assignment problem.

## 6 Source assignment problem

In an EPM-compressed form C=R#t, each pointer may be associated with a substring that occurs several times in *R*. We name such occurrences as sources. A source (*pos_i_*, *len_i_*) is assigned to a pointer *p* if $p=(pos_{i},len_{i})$.

In the EPM formulation, assigning different sources to a pointer does not change the size of the compressed string. However, the assignment of sources may change the number of nodes. According to the two-pass CtoG algorithm, the number of cuts made in the reference is equal to the number of distinct pointer boundaries. Therefore, the choice of sources is directly related to the number of nodes in the graph. An example is illustrated in Figures 2 and 4. The last phrase, (7, 2), is associated with two sources, (3, 2) and (7, 2). If we assign (3, 2) to the phrase, which is different from the case in Figure 2, the number of nodes created will be 5. Otherwise, six nodes will be created as in Figure 4.

Given an EPM-compressed form and the set of sources corresponding to each pointer, if we can assign sources such that the total number of unique pointer boundaries is minimized, we can reduce the size of the created graph. We formulate the source assignment problem and present an integer linear programming (ILP) solution for the optimal source assignment during genome graph construction.

Problem 3 (Source assignment problem). *Given a collection of sources sets* $S=\{S_{1},S_{2},\ldots ,S_{n}\}$*, where S_i_ denotes the set of sources for a unique pointer i, find a set of sources* S′  *such that for all* $S_{i},\;S_{i}\cap S\prime \neq \emptyset$  *and* $\left|\underset{s_{m}\in S\prime }{\cup }\left\{b_{m},e_{m}\right\}\right|$  *is minimized, where b_m_, e_m_ are boundaries of source m.*

In this problem, we choose one source for each pointer such that the union of boundaries $\left\{b_{m},e_{m}\right\}$ of each chosen source $s_{m}=(pos_{m},len_{m})$ is minimized. As a reminder, *b_m_* = *pos_m_* and $e_{m}=pos_{m}+len_{m}$. For convenience, we denote the union of boundaries in a source set *S* by $\underset{B}{\cup }\left\{S\right\}$, which is equivalent to $\underset{s_{m}\in S}{\cup }\left\{b_{m},e_{m}\right\}$.

The formulation of the source assignment problem is similar to the hitting set problem in that it chooses the minimum number of positions to hit every pointer. However, the objective is indirectly related to the number of the chosen sources, and the sources and pointers are defined in a string context. The hardness of the source assignment problem is open due to these differences from the setting of the hitting set problem. Still, the similarities to the hitting set problem lead to the formulation of an integer linear programming solution.

### 6.1 Integer linear programming formulation

The objective of the ILP is to minimize the number of cuts made in the reference, where each cut is made at the boundaries of chosen sources. For each chosen source $s=(pos_{i},len_{i})$, a cut is placed at positions *pos_i_* and $pos_{i}+len_{i}$, which are left and right boundaries of *s*.

We first construct a set of integers *I* that is the union of all source boundaries. Create a binary variable *x_p_* for each p∈I. *x_p_* is set to one if a cut is made at position *p*.

We create a binary variable $y_{s_{i}}$ for each source $s_{i}=(pos_{i},len_{i})$ that indicates whether the source is chosen. We create a constraint (Inequality 7) that at least one source is chosen from each set. We create another pair of constraints (Inequality 8) that ensures that if a source is chosen, two cuts are made at its left (*pos_i_*) and right ($pos_{i}+len_{i}$) boundaries. This leads to the ILP:

(6) $$\min\sum_{p\in I}x_{p}$$

 

(7) $$\text{subject}\;\text{to}\;\;\;\;\sum_{s_{j}\in S_{i}}y_{s_{j}}\geq 1\;\;\;\;\;\;\;\;\;\;\;\;\;\;\forall S_{i}\in S$$

 

(8) $$y_{s_{j}}\leq \min\{x_{pos_{j}},x_{pos_{j}+len_{j}}\}$$

 

(9) $$x_{p},y_{s_{j}}\in \{0,1\}$$

### 6.2 Pruning to reduce the number of sources

In practice, a pointer with a short length may correspond to a large number of sources. For example, a pointer with length one may correspond to |R|/4 sources, where *R* is the reference string and when the alphabet size is 4. This could result in a huge number of variables in the ILP formulation and would hinder its practicality significantly.

To address this, we preprocess the sources as follows. If a source does not intersect with any other sources of different pointers, we eliminate the source from the source set unless it is the only source of a pointer. We name the eliminated sources isolated sources. Removing such sources does not affect the optimality of the solution.

Lemma 4. *If a set of sources, S, that satisfies the constraints of the source assignment problem, includes an isolated source s, it is possible to find a set of sources* S′  *with equal or lower objective value that does not include s.*

Proof. Let the pointer for the isolated source be *p* and the source set of *p* be *S_p_*. Since *s* is an isolated source, there must be at least another source s′ in *S_p_*. If s′ also does not intersect with any other sources in *S*, $S\prime =|\underset{B}{\cup }\{(S\setminus s)\cup s\prime \}|=|\underset{B}{\cup }\left\{S\right\}|$. Otherwise, if s′ intersects with some sources in *S*, this means that the union of source boundaries is reduced by at least 1 if we replace *s* with s′, i.e. $S\prime =|\underset{B}{\cup }\{(S\setminus s\prime )\cup s\}|\leq |\underset{B}{\cup }\left\{S\right\}|-1$. Therefore, excluding all isolating sources during preprocessing does not affect the optimality of the solution. □

## 7 RLZ graph

As a proof-of-concept that constructing a genome graph using a compression scheme results in small graphs, we implement the graph construction algorithm based on an EPM compression scheme algorithm, RLZ. Given a reference string *R*, the RLZ algorithm (Kuruppu *et al.*, 2010), introduced in Section 2.4, seeks to greedily produce a compressed form of *R* where all pointers in *R* are right-maximal. We name the right-maximal pointers phrases. RLZ factorization in this manuscript is done on the compressed suffix array in the SDSL C++ library (Gog *et al.*, 2014).

We apply the two-pass CtoG algorithm described in Section 4.1 to construct a RLZ-Graph. We merge the parallel edges in the implementation as it is the common practice in genome graph storage.

An example of RLZ-Graph is shown in Figure 4. The RLZ-Graph is constructed based on the RLZ factorization in Figure 2, where the reference string is *R*=*ATCGATAGA*, the input string is *T*=*TCGAGATGA* and the factored phrase sequence is t=(1,4),(3,3),(7,2). The nodes are produced by segmenting *R* according to the boundaries of sources assigned to phrases in *t*.

In the implementation of RLZ-Graph, we build a bidirected graph where each node can be traversed in forward and reverse directions. For each node v=(pos,len), *pos* is referred to as the head of the node and *pos* + *len* is referred to as the tail. If a node is traversed in reverse direction, its label is denoted as $\hat{\ell }(v)$, which is equal to the reverse complement of ℓ(v). This technique is useful in genomic sequences that underwent structural variations such as inversions, where the entire genomic segment is replaced by its reverse complement due to a double-strand break. During the construction of the RLZ-Graph, we use a modified reference sequence *R* by concatenating the reference genome of the organism of interest with its reverse complement. Before the source assignment step, we mark each source as reversed if it is located on the reverse complement half of *R* and translate its boundary positions to the forward half. After the source assignment step, we mark a pointer as reversed if it is assigned a reversed source. When we add edges, if we encounter a reverse pointer p=(pos,len), we add an edge directing to the tail of the node $v_{i}=(pos_{i},len_{i})$ and an edge directing from the head of the node $v_{j}=(pos_{j},len_{j})$, where *pos_j_* = *pos* and $pos_{i}+len_{i}=\mathrm{pos}+\mathrm{len}$.

### 7.1 Different source assignment heuristics

Aside from the ILP solution to the source assignment problem, sources are chosen by other heuristics in literature regarding RLZ factorization (Kuruppu *et al.*, 2011). Specifically, from the source set corresponding to a phrase, the leftmost source on the reference string is chosen (Left), or the lexicographically smallest source is chosen (Lex). A source $s_{i}=(pos_{i},len_{i})$ is to the left of source $s_{j}=(pos_{j},len_{j})$, or $s_{i}{<}_{\mathrm{left}}s_{j}$, if *pos_i_* < *pos_j_*. A source *s_i_* is lexicographically smaller than *s_j_*, or $s_{i}{<}_{\mathrm{lex}}s_{j}$, if $R[pos_{i}:|R|-1]<R[pos_{j}:|R|-1]$ given a reference string *R*.

In the implementation of RLZ-Graph, the phrase is assigned to the lexicographically smallest source during RLZ factorization. After that, we re-assign the leftmost source to each phrase in order to construct a smaller genome graph, which is the default behavior of the RLZ-Graph software. We discuss in the Supplementary material the performance of various source assignment heuristics to reduce the number of nodes in the graph (Supplementary Section S3).

## 8 Experimental results

We ran all our experiments on a server with 24 cores (48 threads) of two Intel Xeon E5 2690 v3 @ 2.60 GHz and 377 GB of memory. The system was running Ubuntu 18.04 with Linux kernel 4.15.0.

In this section, we compare the size of the colored compacted de Bruijn graphs (Iqbal *et al.*, 2012) and variation graphs (Garrison *et al.*, 2018) with that of RLZ-Graphs on human genomic sequences. There are many genome graph construction methods. However, we are mainly concerned with the methods that are based on complete genome sequences as input and generate genome graphs that guarantee exact reconstruction of input sequences. Specifically, we focus on comparing to colored compacted de Bruijn graphs. While there have been many graph construction algorithms for building colored de Bruijn graphs, the graph structure of ccdBG remains the same in these algorithms despite the different approaches to store the reconstruction paths as identifiers in each node.

The comparisons made in this section only concern the graph structure, which includes the nodes, edges and the node labels.

### 8.1 Performance of RLZ-Graph compared to the colored compacted de bruijn graphs

We use Bifrost (Holley and Melsted, 2020) to construct the ccdBG. The genome graphs constructed include nodes, labels of nodes and edges, and are stored in graphical fragment assembly (GFA) format (Li, 2016). In a GFA file, the nodes of a graph are stored as a list of pairs of node identifiers and labels, and edges are stored as a list of pairs of node identifiers. Same as the RLZ-Graph, the graph constructed by Bifrost is bidirected and does not contain parallel edges. The RLZ-Graph produced in this section does not use the ILP solution to assign sources due to time and memory concerns. Instead, we adopt the leftmost heuristic, where the leftmost source is assigned to each phrase.

We build the graphs on all human chromosomes and show the results on chromosome 1 in this section (see Supplementary Figs S1–S3 for the rest of the chromosomes). The genomes we use are from the 1000 Genomes Project phase 3 (1000 Genomes Project Consortium, 2015). In each experiment, we randomly choose 5, 25, 50, 75 and 100 samples and generate their genomic sequences on all chromosomes using the consensus command from bcftools (Li, 2011). We construct the ccdBG with Bifrost and RLZ-Graph using the sample sequences and the reference hg37. Hg37 is also used as the reference string during RLZ factorization. We vary the *k*-mer sizes used for Bifrost and report the sizes of graphs with k= 31, 63 and 127. The default choice of *k* of Bifrost is 31. We repeat each experiment five times.

**Fig. 5:**
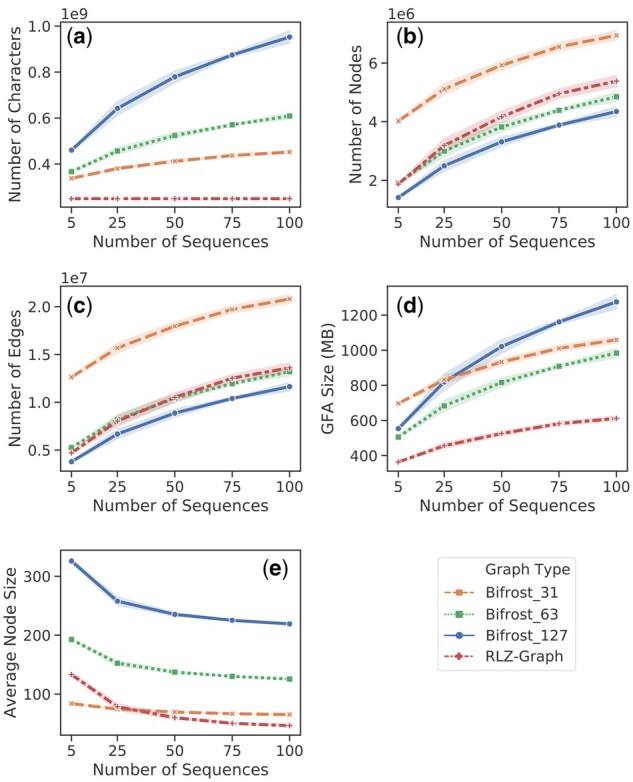
Comparison between RLZ-Graph and ccDBG constructed by Bifrost with *k *=* *31, 63 and 127 on human chromosome 1 sequences. (**a**) Total number of characters in the node labels. (**b**) Number of nodes. (**c**) Number of edges. (**d**) Size of the GFA file that stores the graph structure and node labels. The shaded region represents the standard deviation across five experiments and each data point in the plots represents the mean across five experiments.

As shown in Figure 5, we compare the graph size in different aspects. From 5 sequences up to 100 sequences, the graph produced by RLZ-Graph is smaller than the graph produced by Bifrost with different choices of *k* under all measures in the figure. The number of total characters in the concatenated node labels are constant in the RLZ-Graph regardless of the increase of the number of sequences because nodes are produced by cutting a reference string (Fig. 5(a)). At 100 sequences, the GFA file that stores the RLZ-Graph is 37% smaller than the GFA file storing the colored de Bruijn graph produced by Bifrost with *k *=* *63 and is 42.2% smaller when *k *=* *31 (Fig. 5(d)). When *k* is smaller, the ccdBG becomes impractical for human chromosomes in terms of running time (Supplementary Section S2). Therefore, we do not include the results of graphs constructed with smaller *k* values in Figure 5.

### 8.2 Comparison between ccdBGs, variation graphs and RLZ-Graphs on HGSVC data

In addition to SNPs, the HGSVC dataset (Ebert *et al.*, 2021) provides the set of large-scale insertion, deletion, inversion and translocation events. To evaluate RLZ-Graph on a more comprehensive set of variants, we ran RLZ-Graph, Bifrost and VGtoolkit (Garrison *et al.*, 2018) on the HGSVC dataset, which contains 32 samples from people in various populations.

While VGtoolkit supports genome graph construction given genomic sequences as input, it constructs the graph by iteratively aligning sequences to the graph (Sirén *et al.*, 2014, 2020; Sirén, 2017), which can be inefficient when the lengths and the number of sequences are large. Therefore, we construct variation graphs based on the set of variants as input using VGtoolkit.

In each experiment, we randomly choose 5, 10, 25, 32 samples and generate their sequences using the consensus command from bcftools. We construct the ccdBG with Bifrost and RLZ-Graph using the sample sequences and the reference hg38. We vary the k-mer sizes used for Bifrost and report the size of graphs with *k *=* *31, 63 and 127. The sizes of graphs on disk are evaluated again using the size of the GFA file. VG format is converted to GFA using vg view command. We repeat each experiment five times.

**Fig. 6:**
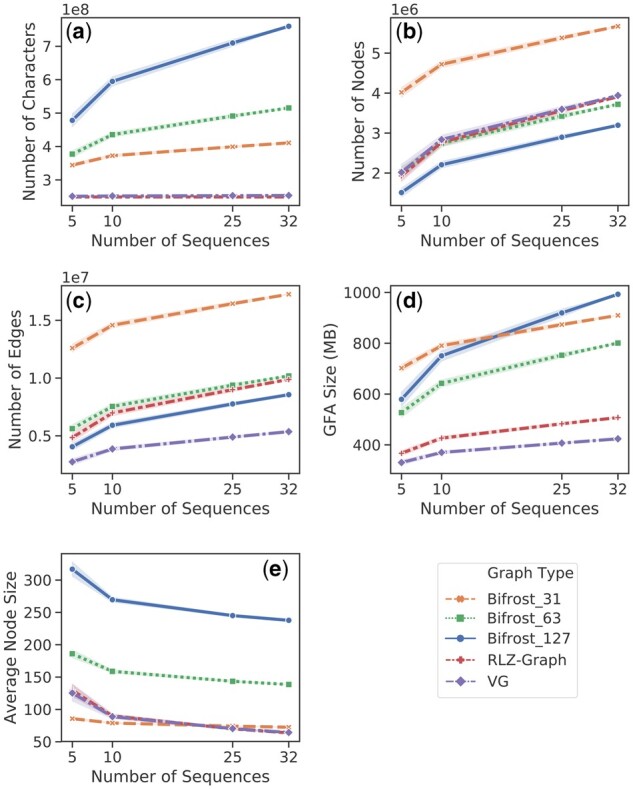
Comparison across RLZ-Graph, ccDBG constructed by Bifrost and variation graph constructed by VGtoolkit. (**a**) The total number of characters in the node labels. (**b**) The number of nodes. (**c**) The number of edges. (**d**) Size of the GFA file that stores the graph structure and node labels. (**e**) The average number of characters in node labels. The shaded region represents the standard deviation across five experiments and each data point in the plots represents the mean across five experiments.

As shown in Figure 6, the sizes of the graphs constructed by Bifrost and RLZ-Graph are similar to those built from the 1000 Genomes Project (Figure 4). RLZ-Graph constructs graphs that have similar sizes as variation graphs, which shows that RLZ-Graph does not depend on preprocessing steps to construct small genome graphs on full-length genomic sequences.

## 9 Discussion

We define the restricted genome graph and formalize the restricted genome graph size optimization problem. The optimization problem balances both the size of the graph structure and the length of the reconstruction paths of sequences stored in the graph, which is similar to the string compression problem. Inspired by the similarity, we present a pair of algorithms that bridge genome graph construction and the EPM model. We prove an upper bound on the size of the genome graph that is constructed based on an optimally compressed string from the EPM model. One key advantage of our graph construction algorithm is that the total number of characters stored in the graph remains constant regardless of the number of sequences stored in the graph, which keeps the space taken by the graph small. Further, since the number of nodes and edges are derived from an already compressed representation of strings, the number of nodes and the number of edges remain small.

Equivalent choices made by data compression algorithms may affect the size of the genome graph differently (Supplementary Section S3). We address this discrepancy by solving the source assignment problem, which is not limited to the RLZ algorithm but can be applied to any EPM-compressed form to reduce the number of nodes and edges. The NP-completeness of the source assignment problem is still open.

As a proof-of-concept that compression-based genome graph construction algorithms can produce small genome graphs, we implement RLZ-Graph based on the RLZ algorithm (Kuruppu *et al.*, 2010). We show that RLZ-Graph can reduce the size of the graph significantly on disk compared to the colored compacted de Bruijn graph.

RLZ-Graph does not depend on hyperparameters or preprocessing steps to construct genome graphs on full-length genomic sequences. The choice of *k*-mer sizes is important in de Bruijn graph construction as it significantly affects the size of the graph. RLZ-Graph removes this dependence on the choice of *k* and produces practical graphs with a smaller size that is scalable to the entire human genome. On the other hand, RLZ-Graph produces graphs with similar sizes to VGtoolkit, even when the genome sequences are not processed by variant callers or sequence aligners.

While existing genome graph indexing methods (e.g. Sirén *et al.*, 2020, 2014; Sirén, 2017) can be applied to RLZ-Graphs for downstream genomics analysis, such as alignment and variant calling, it may be more efficient to use an index specialized for RLZ factorization. There has been a line of work focusing on fast sequence query given a string compressed by the RLZ algorithm (Do *et al.*, 2014; Gagie *et al.*, 2012; Navarro and Sepúlveda, 2019). It is possible to extend these text indices to graph indices that enable faster sequence query.

This work is an initial investigation into the connection between genome graph construction and string compression. We show that using compression algorithms, we can build small genome graphs efficiently, which opens up the possibilities in future research in adapting other data compression schemes to genome graph construction.

## Funding

This work was supported in part by the Gordon and Betty Moore Foundation’s Data-Driven Discovery Initiative [GBMF4554 to C.K.], by the US National Institutes of Health [R01GM122935], and the US National Science Foundation [DBI-1937540]. The Carnegie Mellon University School of ComputerScience Sansom graduate fellowship for computational cancer research to Y.Q.

*Conflict of Interest*: C.K. is a co-founder of Ocean Genomics, Inc.

## Supplementary Material

btab281_Supplementary_Data
