# Supplementary material for: Constructing small genome graphs via string compression
Source: Bioinformatics. 2021 Jul 12;37(Suppl 1):i205–13. doi: 10.1093/bioinformatics/btab281 (PMC8275343; doi:10.1093/bioinformatics/btab281)
Supplement: btab281_Supplementary_Data [file btab281_supplementary_data.zip › Files/Kingsford.220.supp.1[AU].pdf]

Supplementary Material for Constructing Small Genome Graphs via String Compression

Yutong Qiu[1] and Carl Kingsford[1,2]

[1] Carnegie Mellon University, Pittsburgh PA, 15213
[2] To whom correspondence should be addressed. Email: carlk@cs.cmu.edu

S1 Proofs of lemmas and theorems in the main text

S1.1 Two-pass "CtoG" algorithm

We state Theorem 1 in Section 4.1 of the main text.

(Main text) Theorem 1. *Given an EPM-compressed form of string T, $C = R\#t$, the* `two-pass CtoG` *algorithm creates a genome graph $G = (V, E, \ell)$ that contains reconstruction paths for R and T.*

Proof. In the second pass of the algorithm, edges are added between the nodes that are the suffix and the prefix of adjacent pointers. Therefore, all pointer adjacencies are represented as edges in the genome graph.

All substrings $R[i : j]$ can be reconstructed from G. If $R[i : j]$ is a substring of a node label, it can be reconstructed from G. If $R[i : j]$ spans two nodes, it spans two nodes connected by a reference edge.

Any substring $T[i : j]$ can be reconstructed from G. Suppose position i lands in the middle of a pointer $p_k = (pos_k, len_k)$, which means that $k \leq i \leq k + len_k - 1$.

1. If $j \leq k + len_k - 1$, which means that $T[i : j]$ is a substring of the string represented by a pointer. Since all pointers in t point to substrings in R and R can be reconstructed from G, a substring of a pointer can be reconstructed.

2. If $j > k + len_k - 1$, which means that $T[i : j]$ spans at least two pointers. From the previous case, we have that $T[i : k + len_k - 1]$ can be reconstructed using nodes and edges in G. Since all adjacencies between two pointers are represented in G, we can apply the analysis to the rest of $T[i : j]$. Therefore $T[i : j]$ can be reconstructed if it spans more than one pointer.

Finally, we show that the created graph is a restricted genome graph. During the second pass, a reference edge is added for each node adjacency within each pointer, and an edge is added for each pointer adjacency. Therefore, each edge is only used once in all of the reconstruction paths.

□

S1.2 Upper-bounds on the size of the restricted genome graphs and the EPM-compressed forms

We prove Lemma 1, Lemma 2 and Lemma 3 from Section 5 of the main text.

(Main text) Lemma 1. *Given an optimally compressed EPM form $C = R\#t$ of text T, the size of the transformed restricted genome graph $G = (V, E, \ell)$, $size(G)$, according to* `two-pass CtoG` *in Section 4.1 has an upper bound:*

$$size(G) \leq |R| \cdot \log|\Sigma| + \min(2n, |R|) \cdot 2\log|R| + (\min(2n, |R|) \cdot |t| - 1 + 2m) \cdot 2\log(\min(2n, |R|)) \quad (1)$$

where n is the number of unique pointers in t.

Proof. The algorithm introduced in Section 4.1 creates nodes by cutting the reference string R according to the boundaries of pointers. Each node is stored as a pointer (pos, len) to R, which takes $2\log|R|$ bits.

The total number of nodes produced by cutting the reference is $\leq \min(2n, |R|)$. The number of cuts introduced by each unique pointer is ≤ 2. The maximum number of nodes given a reference string R is $|R|$. Therefore, the space to store all the nodes is $\leq \min(2n, |R|) \cdot 2\log|R|$.

The total number of edges, including reference and non-reference edges, in a restricted genome graph is $\leq \min(2n, |R|) \cdot |t| - 1$. After the first pass of `two-pass CtoG`, the interval corresponding to each pointer may be cut into several nodes. Let the average number of nodes contained in each pointer's interval be $a \leq |V| \leq \min(2n, |R|)$. The average number of reference edges within each pointer is then $a - 1$, and the total number of edges within pointers is $(a-1) \cdot |t|$. The total number of edges between pointers is $|t| - 1$. Since T represents m sequences, we have $2m$ additional edges directing to and from the sink and source. Together, the number of edges in the reconstruction path is $a \cdot |t| - 1 \leq \min(2n, |R|) \cdot |t| - 1 + 2m$.

The size of the genome graph is then:

$$\begin{aligned} size(G) &= |R| \cdot \log|\Sigma| + |V| \cdot 2\log|R| + |E| \cdot 2\log|V| \\ &\leq |R| \cdot \log|\Sigma| + \min(2n, |R|) \cdot 2\log|R| + (\min(2n, |R|) \cdot |t| - 1 + 2m) \cdot 2\log(\min(2n, |R|)). \end{aligned}$$

□

(Main text) Lemma 2. *Given a restricted genome graph, $G = (V, E, \ell)$, constructed from an optimally compressed EPM form $C = R\#t$, the size of the genome graph, $G' = (V, E', \ell)$, produced by merging parallel edges in G has an upper bound:*

$$size(G') \leq |R| \cdot \log|\Sigma| + \min(2n, |R|) \cdot 2\log|R| + (\min(2n, |R|) + |t| - 1 + 2m) \cdot 2\log(\min(2n, |R|)), \quad (2)$$

where n is the number of unique pointers in t.

Proof. Merging parallel edges does not change the number of nodes and the concatenation of node labels.

The number of reference edges in G' is equal to $|V|-1$, as the nodes are produced by cutting the reference string.

The number of forward and backward edges in G is equal to $|t|-1$, and the number of forward and backward edges in G' is $\leq |t|-1$ due to parallel edge merging. According to `two-pass CtoG`, since C is optimal, only a forward or a backward edge can be added for each pair of adjacent pointers in t during the second pass. Suppose two adjacent pointers, $p_1 = (pos_1, len_1)$ and $p_2 = (pos_2, len_2)$, result in a reference edge, which means that $pos_2 = pos_1 + len_1$, the two pointers can be merged into $p_3 = (pos_1, len_1 + len_2)$. Merging two pointers reduces the size of C, which contradicts the assumption that the size of C is optimal.

Together, the space to store all the edges in G' is $\leq (|V| + |t| - 1) \cdot 2\log\min(2n, R) \leq (\min(2n, |R|) + |t| - 1 + 2m) \cdot 2\log\min(2n, |R|)$.

Therefore, the size of the genome graph G' after merging the parallel edges in G is:

$$
\begin{aligned}
|G'| &= |R| \cdot \log|\Sigma| + |V| \cdot 2\log|R| + |E'| \cdot 2\log|V| \\
&\leq |R| \cdot \log|\Sigma| + \min(2n, |R|) \cdot 2\log|R| + (\min(2n, |R|) + |t| - 1 + 2m) \cdot 2\log(\min(2n, |R|)).
\end{aligned}
$$

□

(Main text) Lemma 3. *Given a restricted genome graph $G = (V, E, \ell)$ of a collection of strings $\mathcal{S}$, the size of the transformed EPM-compressed form of the concatenated strings in $\mathcal{S}$, $C = R\#t$ according to `GtoC` described in Section 4.1 has an upper bound:*

$$size(C) \leq |R| \cdot \log|\Sigma| + |E| \cdot \log\binom{|V|+1}{2} + 2\binom{|V|+1}{2}\log|R|, \tag{3}$$

where R is a string formed by concatenating all node labels.

Proof. Let the set of paths corresponding to the set of strings $\mathcal{S}$ be $\mathcal{P} = \{P_1, P_2, ..., P_m\}$, where m is the number of strings in $\mathcal{S}$. Since G has an optimal size, the number of edges in G is exactly $|E| = \sum_{i\in[1,m]}(|P_i|-1)+2m = \sum_{i\in[1,m]}|P_i|+m$, where E includes reference, forward and backward edges. Note that if an edge does not belong to any path in $\mathcal{P}$, it can be eliminated in the graph, which results in a smaller restricted genome graph.

According to `GtoC`, the pointers are created by either directly converting a node or merging two or more nodes connected by a reference edge in a path $P \in \mathcal{P}$. The number of pointers in t, or $|t|$, is less than $|E|$.

Given $|V|$ nodes, the reference constructed by concatenating all node labels contains $|V|+1$ cut positions including the positions before $R[0]$ and after $R[|R|-1]$. From these cut positions, we

can produce at most $\binom{|V|+1}{2}$ pointers by selecting two positions as boundaries of a pointer. Let the total number of unique pointers be n. Then $n \leq \binom{|V|+1}{2}$.

Together, the size of the EPM-compressed form is:

$$size(C) = |R| \cdot \log|\Sigma| + |t| \cdot log\ n + n \cdot 2\log|R| \quad (4)$$

$$\leq |R| \cdot \log|\Sigma| + |E| \cdot \log\binom{|V|+1}{2} + \binom{|V|+1}{2} \cdot 2\log|R|. \quad (5)$$

□

S2 Running time and peak memory used by Bifrost and RLZ-Graph on 1000 Genome Project dataset

The average wall-clock running time and resident set size (RSS) of RLZ-Graph and Bifrost [2] with $k = 31,\ 63$ and 127 on chromosome 1 are reported in Table 1 and 2. It takes RLZ-Graph around 2.5 hours to build a graph with 100 chromosome 1 sequences. The running time includes the time to do RLZ factorization. In all experiments, Bifrost is run in parallel in 20 threads while RLZ-Graph is run in a single thread.

The RLZ-Graph implementation is not optimized and not parallelized compared to the implementation of Bifrost. Still, the running time of RLZ-Graph is on a similar scale compared to Bifrost. As RLZ-Graph is not optimized for memory usage, the peak memory used by RLZ-Graph grows linearly in the number of input sequences. A future direction would be to improve the implementation of RLZ-Graph by parallelizing the RLZ factorization step.

When $k = 15$, the size of the GFA file that stores the ccdBG is 15 gigabytes for 5 chromosome 1 sequences and the running time of Bifrost is around 8 hours, while the running time is 396 seconds for $k = 31$. Both the size of the graph and the running time is impractical compared to other k values. When $k = 3$, the size of the GFA file is 4.2 kilobytes for 5 chromosome 1 sequences with 32 nodes and 127 edges, and the running time of Bifrost is around 2.5 hours. Although the graph is small, it is similar to the G_4 solution to the genome graph size optimization problem, where the length of the reconstruction path is approximately the same as the original string.

Table 1. Average wall-clock running time of RLZ-Graph and Bifrost with different k values on chromosome 1 sequences.

Number of sequences	5	25	50	75	100
**RLZ-Graph time (s)**	1031	2707	4758	6872	9136
**Bifrost k=31 time (s)**	396	656	986	1542	1852
**Bifrost k=63 time (s)**	280	510	793	1126	1332
**Bifrost k=127 time (s)**	412	733	1098	1443	1744

Table 2. Average resident set size of RLZ-Graph and Bifrost with different k values on chromosome 1 sequences.

Number of sequences	5	25	50	75	100
**RLZ-Graph RSS (MB)**	8562	13316	19258	25918	33762
**Bifrost k=31 RSS (MB)**	4450	4716	7510	5019	7550
**Bifrost k=63 RSS (MB)**	6904	6961	7079	7528	7567
**Bifrost k=127 RSS (MB)**	6949	7059	7655	7727	7803

S3 Performance of various source assignment heuristics on *E. coli* genomes

In the EPM formulation, assigning different sources to a pointer does not change the size of the compressed string, but may affect the number of nodes (main text Section 6).

Aside from the ILP solution (main text Section 6.1) to the source assignment problem, sources are chosen by other heuristics in literature regarding RLZ factorization [3]. Specifically, from the source set corresponding to a phrase, the leftmost source on the reference string is chosen (Left), or the lexicographically smallest source is chosen (Lex). A source $s_i = (pos_i, len_i)$ is to the left of source $s_j = (pos_j, len_j)$, or $s_i <_{left} s_j$, if $pos_i < pos_j$. A source s_i is lexicographically smaller than s_j, or $s_i <_{lex} s_j$, if $R[pos_i : |R| - 1] < R[pos_j : |R| - 1]$ given a reference string R. We compare the number of nodes eliminated by various source assignment heuristics on *E. coli* genomes.

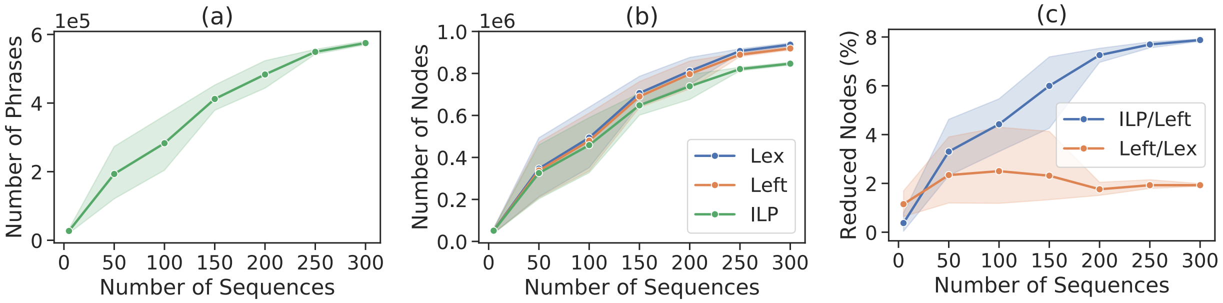

Fig. S1. Performance of heuristics solving the source assignment problem. (a) The number of phrases. (b) The number of nodes. (c) Percentage of nodes reduced using the leftmost heuristic and the ILP solution during the source assignment step. The shaded area in the plots represents the standard deviation across 5 experiments and each data point in the plots represents the mean across 5 experiments. Lex: lexicographical heuristic. Left: leftmost heuristic. ILP: ILP solution.

We obtain 300 genomic assemblies of *E. coli* O157 strain from Genbank [1]. In each experiment, we randomly permute the 300 sequences and construct the RLZ-Graph on the first 50, 100, 150, 200, 250, 300 sequences. The first sequence in the randomly permuted 300 sequences is used as the reference string. We repeat each experiment 5 times.

In Figure S1(a), we show the rate at which the number of phrases produced by the RLZ factorization increases as the number of sequences increases. In Figure S1(b), we show the number of nodes produced due to different source assignment strategies. The ILP solution has the best performance and results in the fewest nodes. The percentage of reduced nodes is around 8% for

300 *E. coli* sequences. As the number of sequences increases, the ILP solution is able to eliminate more nodes compared to the heuristic that always chooses the leftmost source (Figure S1(c)). The percentage of eliminated nodes is calculated as $1-(|V|_{ILP}/|V|_{Left})$ and $1-(|V|_{Left}/|V|_{Lex})$, respectively.

Solving the source assignment problem prior to graph construction reduces the number of nodes by around 8%. Although it is a relatively small percentage, when dealing with very large genome graphs, it translates into substantial space-saving.

S4 Comparison between ccdBGs and RLZ-Graphs on human chromosomes 2–22

We compare the sizes of genome graphs constructed on human chromosomes 2–22 by RLZ-Graph and Bifrost in Figures S2-S4. The experiment settings are the same as in Section 8.1 of the main text. Bifrost is run with $k=31$, which is the default setting.

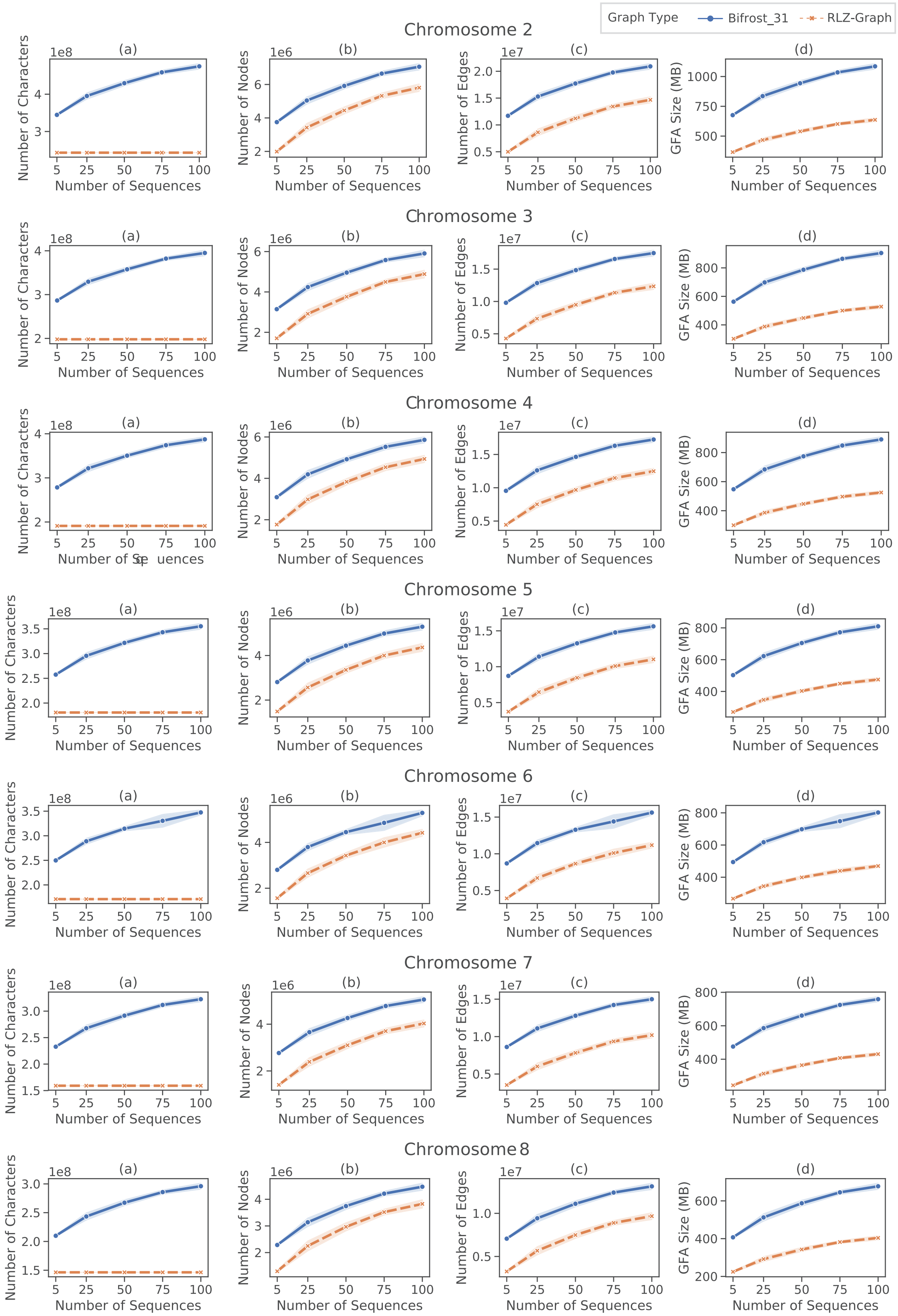

Fig. S2. Comparison between RLZ-Graph and ccdBG constructed by Bifrost with $k = 31$ on human chromosomes 2–8. (a) Total number of characters in the node labels. (b) Number of nodes. (c) Number of edges. (d) Size of GFA file that stores the graph structure and node labels.

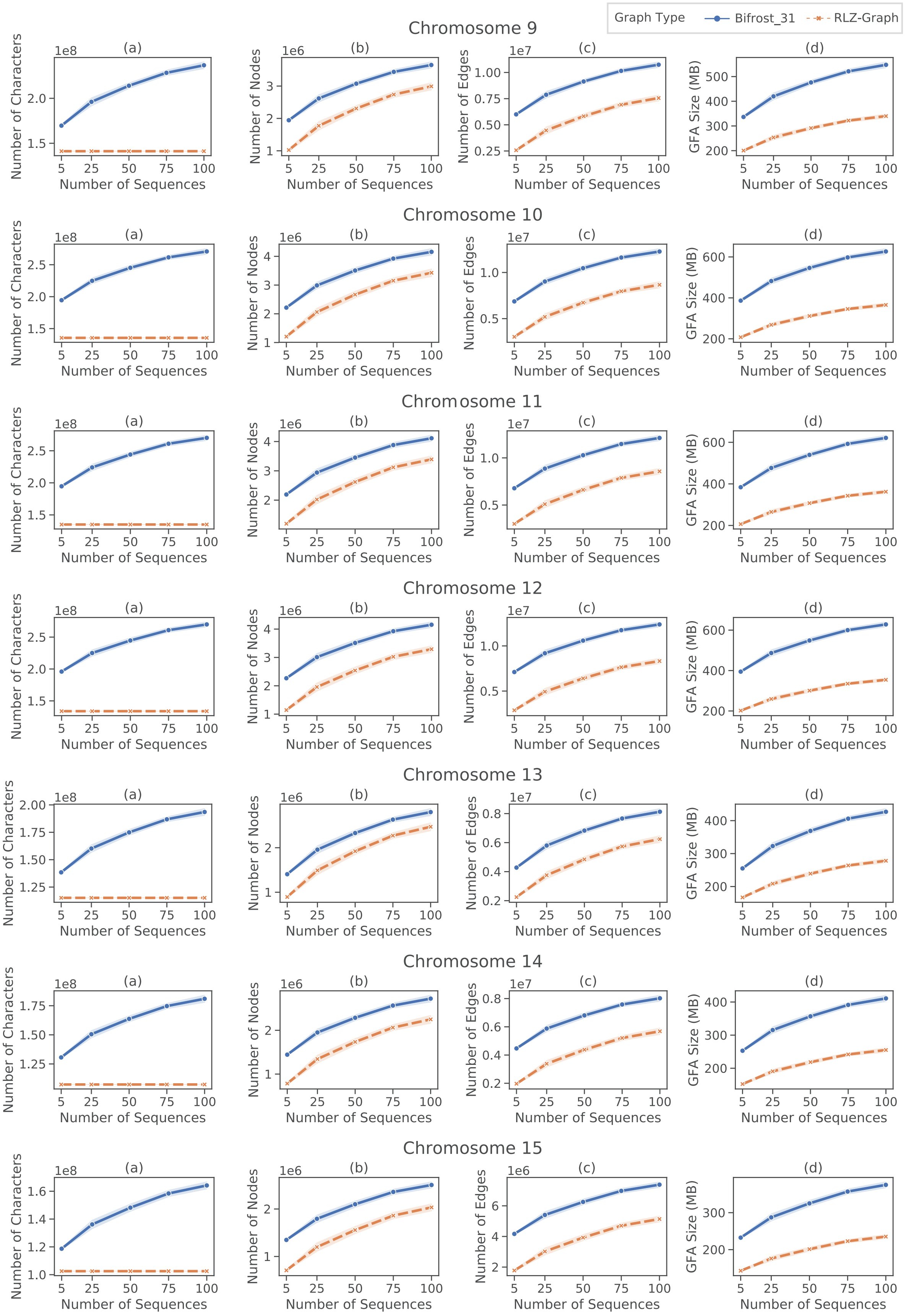

Fig. S3. Comparison between RLZ-Graph and ccdBG constructed by Bifrost with $k = 31$ on human chromosomes 9–15. (a) Total number of characters in the node labels. (b) Number of nodes. (c) Number of edges. (d) Size of GFA file that stores the graph structure and node labels.

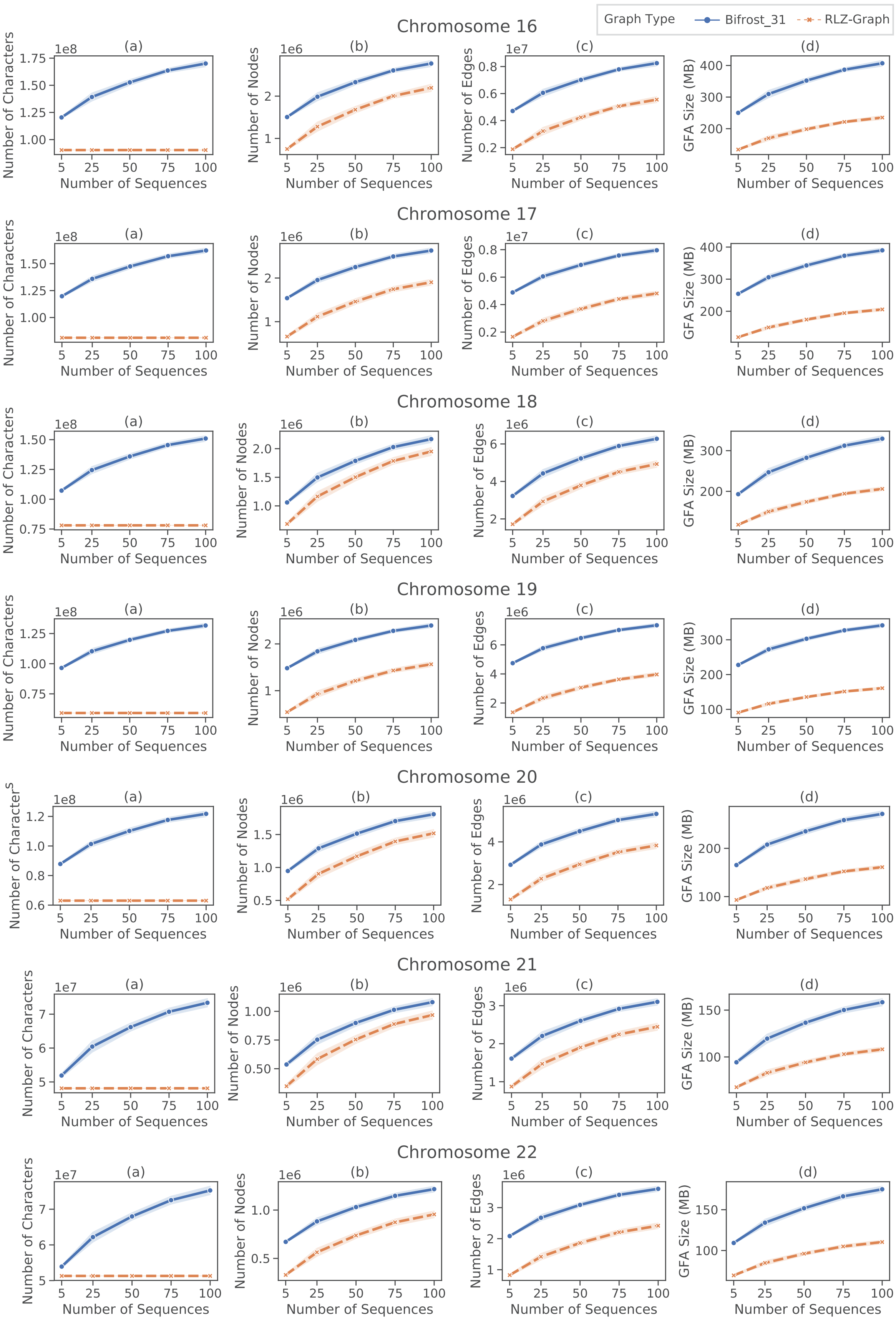

Fig. S4. Comparison between RLZ-Graph and ccdBG constructed by Bifrost with $k = 31$ on human chromosomes 16–22. (a) Total number of characters in the node labels. (b) Number of nodes. (c) Number of edges. (d) Size of GFA file that stores the graph structure and node labels.

References

1. Karen Clark, Ilene Karsch-Mizrachi, David J Lipman, James Ostell, and Eric W Sayers. GenBank. *Nucleic Acids Research*, 44(D1):D67–D72, 2016.

2. Guillaume Holley and Páll Melsted. Bifrost: highly parallel construction and indexing of colored and compacted de Bruijn graphs. *Genome Biology*, 21(1):249–269, 2020.

3. Shanika Kuruppu, Simon J Puglisi, and Justin Zobel. Optimized relative Lempel-Ziv compression of genomes. In *Proceedings of the Thirty-Fourth Australasian Computer Science Conference-Volume 113*, pages 91–98. Australian Computer Society, Inc., 2011.
